# Effects of Adding P2Y12 Inhibitor to Anti-staphylococcal Therapy on Bacterial Clearance in Patients With Staphylococcus aureus Bacteremia

**DOI:** 10.7759/cureus.71984

**Published:** 2024-10-21

**Authors:** Nobuaki Mori, Yuichi Shibata, Wataru Ohashi, Jun Hirai, Nobuhiro Asai, Hiroshige Mikamo

**Affiliations:** 1 Department of Clinical Infectious Diseases, Aichi Medical University, Nagakute, JPN; 2 Department of Infectious Diseases, Aichi Medical University Hospital, Nagakute, JPN; 3 Division of Biostatistics, Clinical Research Center, Aichi Medical University, Nagakute, JPN

**Keywords:** antimicrobial therapy, blood culture, microbiological clearance, p2y12 inhibitor, staphylococcus aureus bacteremia

## Abstract

Background: *Staphylococcus aureus* (*S. aureus*) bloodstream infection (SAB) remains a major clinical challenge despite appropriate antimicrobial therapy. Recent studies have suggested the potential benefits of P2Y12 inhibitors in SAB treatment, but controversy persists regarding their optimal use. Moreover, the effects of P2Y12 inhibitors in Japanese patients remain unclear. This study aimed to evaluate the effects of using P2Y12 inhibitors before admission on outcomes in Japanese patients with SAB.

Methods: This retrospective study involved Japanese patients aged 18 years and older with *S. aureus*-positive blood cultures at Aichi Medical University Hospital in Japan from March 2015 to April 2023. The primary outcome was the rate of persistent bacteremia among patients with follow-up blood cultures within seven days. The secondary outcomes were 30-day and in-hospital mortality rates and bleeding events during hospitalization.

Results: During the study period, 238 patients with SAB (19 P2Y12 inhibitor users and 219 non-users) were enrolled. The median age was 77 years, and 38.7% of the patients were female individuals. The overall rate of methicillin-resistant *S. aureus* as a percentage of *S. aureus* was 37.9%, with no significant difference between the groups. The rate of persistent bacteremia was significantly lower in P2Y12 inhibitor users than in non-users (0% vs. 20.6%, p=0.03). The Kaplan-Meier survival curve for the 30-day and in-hospital mortality rates showed no significant difference between the groups (log-rank test, p=0.25 and p=0.20, respectively). No bleeding events occurred in either group.

Conclusions: This retrospective study suggests that Japanese patients with SAB using P2Y12 inhibitors prior to admission as adjunctive therapy have a higher rate of microbiological clearance than non-users. However, further prospective studies are needed to determine the benefits of adding P2Y12 inhibitors to standard antimicrobial therapy for SAB.

## Introduction

*Staphylococcus aureus* (*S. aureus*) bacteremia infection (SAB) is a formidable clinical challenge associated with high morbidity and mortality. A systematic review of SAB showed that the mortality rate was more than 25% at three months [1]. A distinctive and disconcerting characteristic of SAB is the persistence of *S. aureus* in the bloodstream, even in the presence of microbiologically appropriate antibiotics [2], which underscores the urgent need for novel therapeutic approaches to enhance the efficacy of existing anti-staphylococcal regimens.

Recent studies have suggested that P2Y12 inhibitors, primarily used as antiplatelet drugs in cardiovascular diseases, may have potential as an adjunct therapy for infections owing to their anti-inflammatory and immunomodulatory effects [3,4]. P2Y12 inhibitors might modulate the expression of inflammation cytokines, chemokines, and growth factors through different cellular and molecular mechanisms [5,6]. In the context of SAB, P2Y12 inhibitors have demonstrated the ability to improve host platelet-mediated killing of *S. aureus* and mitigate *S. aureus*-induced thrombocytopenia, likely by preventing α-toxin-related desialylation [4,7]. While these findings are promising, most studies on SAB have been conducted predominantly in Caucasian populations. A retrospective study at Veterans Affairs in the United States in 2022 showed reduced mortality rates among clopidogrel users with SAB but found no difference in microbiological clearance [8]. A case report showed that additional ticagrelor administration effectively cleared persistent bacteremia [9]. These results highlight the need for further research into the mechanisms and timing of P2Y12 inhibitor effects. The efficacy of P2Y12 inhibitors in SAB treatment may be influenced by genetic factors affecting drug metabolism. P2Y12 inhibitor activity is affected by cytochrome (CYP) metabolism. There is a major gap in the literature regarding the efficacy and safety of P2Y12 inhibitors in populations with different genetic backgrounds, particularly those with a high prevalence of CYP2C19 polymorphisms. The frequency of CYP2C19 polymorphisms in Asians is higher than that in Western people [10]. These genetic variations are associated with altered metabolism of P2Y12 inhibitors. For example, the effectiveness of clopidogrel in Japanese individuals is weaker than that in Western people [11,12].

This study aimed to evaluate the effects of P2Y12 inhibitor use before admission on microbiological clearance and clinical outcomes in Japanese patients with SAB, focusing on persistent bacteremia, mortality, and potential adverse events such as bleeding.

## Materials and methods

Patient selection

This study used a retrospective cohort design and involved the use of medical records of patients diagnosed with SAB between March 2015 and April 2023 at Aichi Medical University Hospital, a 900-bed tertiary care hospital located in Aichi, Japan.

This study involved patients 18 years or older with a positive blood culture test for *S. aureus*. The exclusion criteria were as follows: (i) cultures of blood samples that were not collected within seven days of the initial positive culture; (ii) patients who died within three days of finding a positive blood culture; (iii) patients clinically determined to have contaminants; (iv) patients who had not received antimicrobials within three days of positive blood culture; and (v) patients who did not meet the criteria for the P2Y12 inhibitor group but have used P2Y12 inhibitors.

We considered patients with a history of P2Y12 inhibitor use for a minimum of 30 days before admission and with continued use during antimicrobial treatment for SAB. P2Y12 inhibitors included clopidogrel, ticlopidine, clopidogrel/aspirin, and prasugrel. P2Y12 inhibitor non-users were defined as patients who did not use any P2Y12 inhibitors before admission until discharge.

The following data were collected from the patients’ electronic medical records: age, sex, body mass index, underlying diseases, history of operation within 30 days, history of receiving antibiotics within 30 days, history of hospitalization within 30 days, community-onset or not, whether the isolate was methicillin-resistant or not, platelet counts at diagnosis, infection site or focus of SAB, intensive care unit admission at the time of SAB diagnosis, Charlson comorbidity index, follow-up blood culture day, and type and duration of antibiotic administration.

Outcomes

The primary outcome evaluated was the persistent bacteremia rate of *S. aureus*. Persistent bacteremia was defined by two or more positive cultures of blood collected within seven days of the first positive blood culture. The secondary outcomes included 30-day mortality and in-hospital mortality rates and complications associated with bacteremia (e.g., osteomyelitis and abscess), as well as bleeding events.

Statistical analyses

Numerical data are expressed as median and interquartile range (IQR). Categorical data are expressed as numbers and percentages. The Mann-Whitney U test was used to compare continuous variables between P2Y12 inhibitor users and non-users, whereas Fisher’s exact test was used to assess categorical variables. Kaplan-Meier analysis was used to calculate the mortality risk and rate and to show the 30-day and in-hospital mortality rates. For univariate analysis, results with p<0.05 were considered significant. All statistical analyses were performed using Stata software (version 14.2; StataCorp, College Station, TX, USA).

Ethical approval and consent to participate

All procedures were performed in compliance with relevant laws and institutional guidelines. Patient anonymity and privacy were maintained, and the Ethics Committee of Aichi Medical University Hospital reviewed and approved the study design (approval number: 2023-622). Furthermore, the need for patient consent was waived owing to the retrospective nature of this study. The online opt-out option was clearly described and made available to all patients.

## Results

A total of 400 patients with *S. aureus*-positive blood cultures were evaluated for eligibility during the study period. Of these, 162 were excluded based on the exclusion criteria mentioned previously as follows: (i) 85 patients, (ii) three patients, (iii) two patients, (iv) one patient, and (v) 71 patients (the numbers in parentheses indicate the criteria). Therefore, 238 patients were enrolled, including 19 P2Y12 inhibitor users and 219 non-users. The P2Y12 inhibitors used were as follows: 17 clopidogrels, one clopidogrel/aspirin combination, and one prasugrel. The demographic and baseline characteristics of the study population are shown in Table 1.

**Table 1:** Patient characteristics among P2Y12 inhibitor users compared with non-users *Mann-Whitney U test, †Fisher’s exact test IQR: interquartile range, MRSA: methicillin-resistant *Staphylococcus aureus*

Variable	All (n = 238)	P2Y12 inhibitors users (n = 19)	P2Y12 inhibitors non-user (n = 219)	p-value
Age (yr), median (IQR)	77 (64–84)	74 (67–83)	78 (64–84)	0.59^*^
Female sex, no. (%)	92 (38.7)	2 (10.5)	90 (41.1)	0.01^†^
Body mass index, median (IQR)	19.8 (17.0–23.3)	21.1 (19.4–22.3)	19.6 (16.6–23.4)	0.16^*^
Isolate rate of MRSA, no. (%)	88 (37.9)	7 (36.8)	81 (37.0)	1.00^†^
Follow-up blood culture day, median (IQR)	4 (3–5)	4 (3–6)	4 (3–5)	0.14^*^
Past history of operation <30 days no. (%)	17 (7.1)	3 (15.8)	14 (6.4)	0.14^†^
Past history receiving antibiotics <30 days no. (%)	61 (25.6)	8 (42.1)	53 (24.2)	0.10^†^
Past hospitalization <30 days no. (%)	17 (7.1)	3 (15.8)	14 (6.4)	0.14^†^
Baseline platelet count (×10^3^), median (IQR)	19.3 (13.4–26.4)	23.0 (17.4–27.4)	19.3 (12.8–26.3)	0.23^*^
Community-onset, no. (%)	119 (50.0)	8 (42.1)	111 (50.7)	0.63^†^
Infection focus, no. (%)				
Catheter-related bloodstream infection	64 (26.9)	5 (26.3)	59 (26.9)	1.00^†^
Skin and soft tissue infection	46 (19.3)	7 (36.8)	39 (17.8)	0.06^†^
Urinary tract infection	12 (5.0)	0 (0)	12 (5.5)	0.61^†^
Endocarditis	10 (4.2)	0 (0)	10 (4.6)	1.00^†^
Pyogenic arthritis	3 (1.3)	0 (0)	3 (1.4)	1.00^†^
Unknown	89 (37.4)	6 (31.6)	83 (37.9)	0.63^†^
Others	14 (5.9)	6 (31.6)	13 (5.9)	1.00^†^
ICU at diagnosis of SAB, no. (%)	20 (8.4)	0 (0.0)	20 (9.1)	0.38^†^
Underlying diseases, no. (%)				
Diabetes mellitus	62 (26.1)	11 (58.9)	51 (23.3)	<0.01^†^
Chronic kidney disease	52 (21.9)	9 (47.4)	43 (19.6)	<0.01^†^
Heart failure	31 (13.0)	3 (15.8)	28 (12.8)	0.72^†^
Ischemic heart disease	19 (8.0)	8 (42.1)	11 (5.0)	<0.01^†^
Peripheral artery disease	10 (4.2)	5 (26.3)	5 (2.3)	<0.01^†^
Chronic liver disease	13 (5.5)	1 (5.3)	12 (5.5)	1.00^†^
Chronic obstructive pulmonary disease	20 (8.4)	1 (5.3)	19 (8.7)	1.00^†^
Cerebrovascular disease	40 (16.8)	6 (31.6)	34 (15.5)	0.10^†^
Solid organ malignancy	61 (25.6)	6 (31.6)	55 (25.1)	0.59^†^
Hematologic malignancy	10 (4.2)	0 (0.0)	10 (4.6)	1.00^†^
Charlson comorbidity index, median (IQR)	2 (1–3)	4 (3–5)	2 (1–3)	<0.01^*^
Length of hospital stay, median (IQR)	39 (22–69)	43 (19–72)	39 (22–69)	0.88^*^
Total duration of administering antibiotics, median (IQR)	20 (14–32)	20 (14–35)	20 (14–32)	0.50^*^
Duration of administering antibiotics from the negative result of blood culture, median (IQR)	14 (10–28)	17 (10–28)	14 (10–28)	0.44^*^
Antibiotics, no. (%)				
Ampicillin	11 (4.7)	0 (0.0)	11 (5.1)	1.00^†^
Ampicillin/sulbactam	72 (30.3)	4 (21.1)	68 (21.1)	0.44^†^
Cefazolin	64 (26.9)	7 (43.8))	57 (26.0)	0.30^†^
Cefmetazole	2 (0.8)	0 (0.0)	2 (0.9)	1.00^†^
Ceftriaxone	11 (4.6)	0 (0.0)	11 (5.0)	1.00^†^
Cefepime	2 (0.8)	0 (0.0)	2 (0.9)	1.00^†^
Piperacillin/tazobactam	9 (3.8)	1 (5.3)	8 (3.7)	0.53^†^
Meropenem	12 (5.0)	2 (10.5)	10 (4.6)	0.25^†^
Vancomycin	23 (9.7)	2 (10.5)	21 (9.6)	1.00^†^
Teicoplanin	35 (14.7)	2 (10.5)	33 (15.1)	1.00^†^
Daptomycin	98 (41.2)	9 (47.4)	89 (40.6)	0.63^†^
Linezolid	12 (5.0)	2 (10.5)	10 (4.6)	0.25^†^
Tedizolid	6 (2.5)	0 (0.0)	6 (2.7)	1.00^†^
Levofloxacin	7 (2.9)	0 (0.0)	7 (3.2)	1.00^†^
Minocycline	13 (4.5)	0 (0.0)	13 (5.9)	0.61^†^
Clindamycin	5 (2.1)	0 (0.0)	5 (2.3)	1.00^†^
Gentamycin	3 (1.3)	2 (10.5)	1 (0.5)	0.02^†^
Amoxicillin	3 (1.3)	1 (5.3)	2 (0.9)	0.22^†^
Cefalexin	21 (8.8)	1 (5.3)	20 (9.1)	1.00^†^
Amoxicillin/clavulanate	11 (4.6)	0 (0.0)	11 (5.0)	1.00^†^
Sulfamethoxazole/trimethoprim	19 (8.0)	1 (5.3)	18 (8.2)	1.00^†^
Fosfomycin	1 (0.4)	0 (0.0)	1 (0.5)	1.00^†^
Rifampin	6 (2.5)	1 (5.3)	5 (2.3)	0.40^†^

The median age was 77 (range: 69-84) years, and 61.3% of the patients (145/238) were men. The prevalence of methicillin-resistant *S. aureus* as a percentage of *S. aureus* was 37.9% overall, with no significant differences between the groups. There were no injecting drug users among the patients. The types of antimicrobials used were similar in both groups. Although Table 1 lists all antibiotics used during the treatment period, antibiotics to which *S. aureus* was susceptible were administered as treatment in all cases. Platelet counts at diagnosis were not significantly different between the groups. The foci of SAB infection were as follows: 26.9% (64/238) catheter-related bloodstream infection, 19.3% (46/238) skin and soft tissue infection, 5.0% (12/238) urinary tract infection, and 4.2% (10/238) infectious endocarditis. The univariate analysis revealed a significantly higher number of male individuals and individuals with diabetes mellitus, chronic kidney disease, ischemic heart disease, peripheral artery disease, and a higher Charlson comorbidity index among P2Y12 inhibitor users. The median follow-up of blood cultures was four (IQR: 3-5) days, and the median duration of antimicrobial therapy was 20 (IQR: 14-32) days.

Table 2 shows the clinical outcomes of P2Y12 inhibitor users compared with non-users. The overall rate of persistent bacteremia was 18.9% (45/389). The rate of persistent bacteremia was significantly lower in P2Y12 inhibitor users than in non-users (0% vs. 20.6%, p=0.03). The 30-day and in-hospital mortality rates were 6.7% (16/238) and 9.2% (22/238), respectively. The 30-day and in-hospital mortality rates were 0% in the P2Y12 inhibitor users. Complications associated with SAB were observed in 20 patients, including seven patients with abscess formation, five patients with osteomyelitis and emboli, two patients with infected aneurysms, and one patient with septic arthritis. No bleeding events were observed in either group during the hospitalization period.

**Table 2:** Clinical outcomes among P2Y12 inhibitor users compared with non-users ^ †^Fisher’s exact test

Variable	All (n = 238)	P2Y12 inhibitor users (n = 19)	Non-users (n = 219)	p-value
Persistent bacteremia, no. (%)	45 (18.9)	0 (0)	45 (20.6)	0.03^ †^
Complication with bacteremia, no. (%)	20 (8.4)	1 (5.3)	19 (8.7)	1.00^ †^
30-day mortality, no. (%)	16 (6.7)	0 (0)	16 (7.3)	0.62^ †^
In-hospital mortality, no. (%)	22 (9.2)	0 (0)	22 (10.1)	0.23^ †^
Bleeding events, no. (%)	0 (0.0)	0 (0)	0 (0)	1.00^ †^

**Figure 1:**
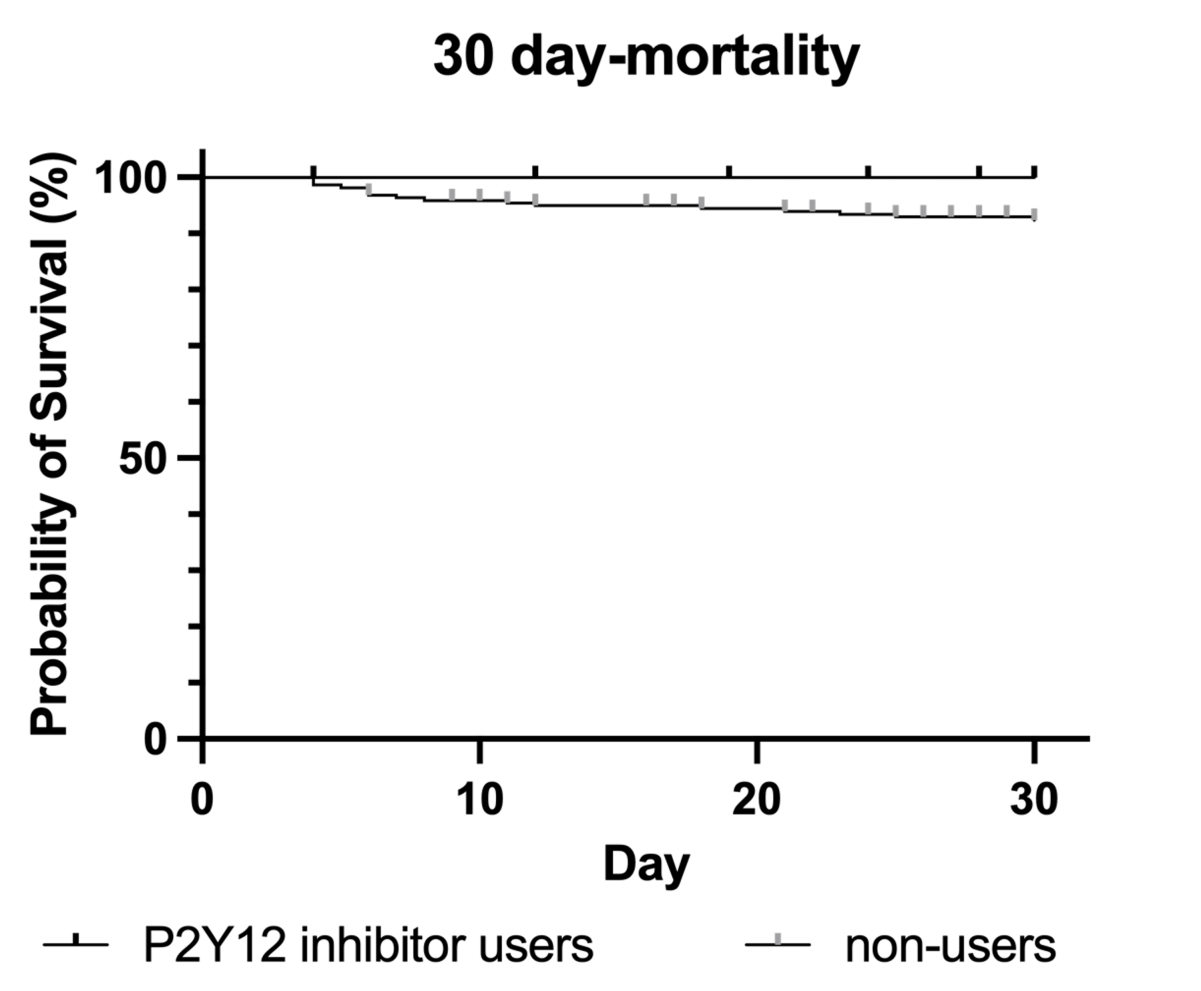
Kaplan–Meier survival curve of the 30-day mortality rates of P2Y12 inhibitor users and non-users from the day of admission

**Figure 2:**
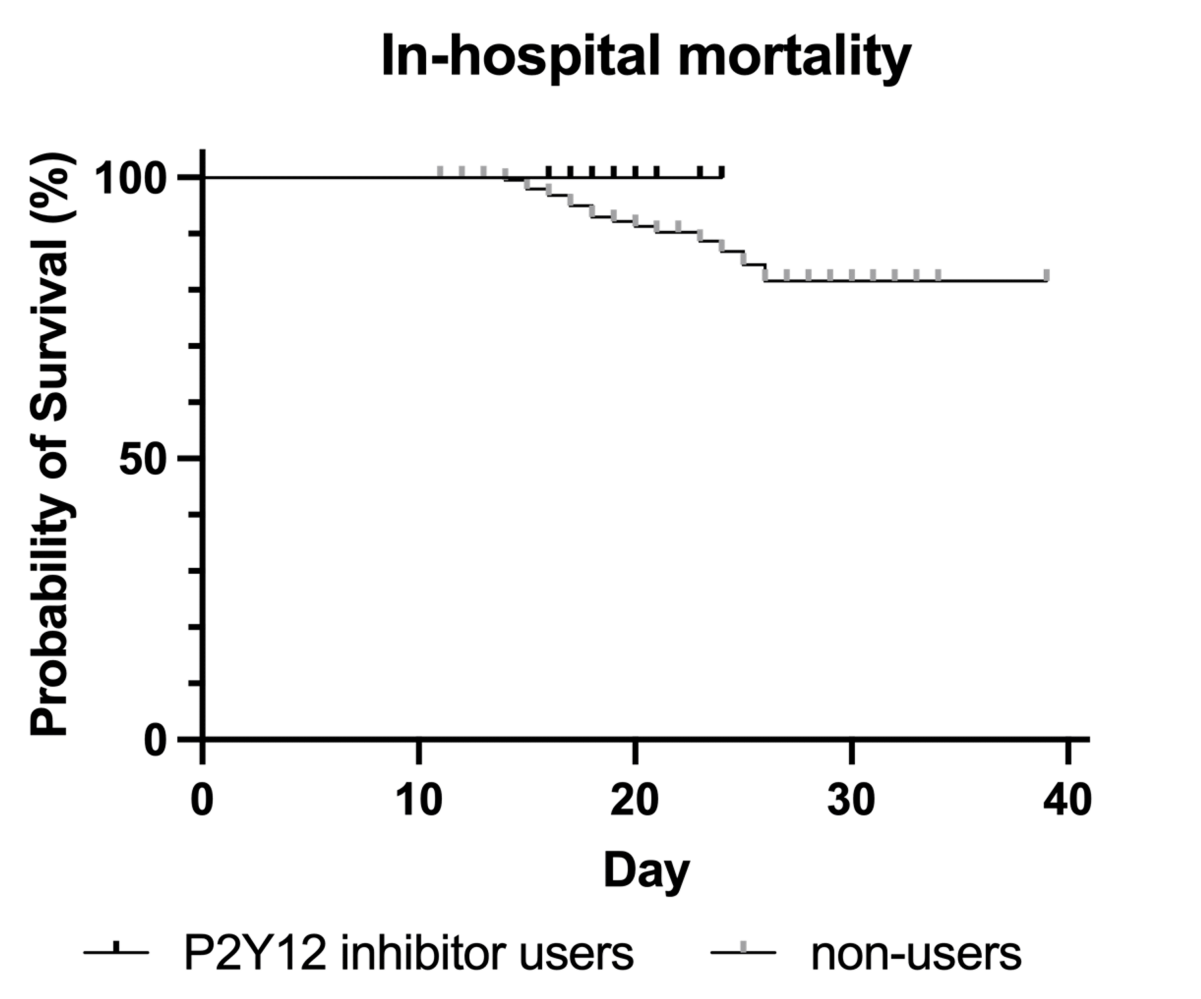
Kaplan–Meier survival curve of the in-hospital mortality rates of P2Y12 inhibitor users and non-users from the day of admission

The Kaplan-Meier survival curve for 30-day and in-hospital mortality showed no significant difference between P2Y12 inhibitor users and non-users (log-rank test, p=0.25 and p=0.20, respectively; Figure 1 and Figure 2).

## Discussion

The findings of this retrospective study of Japanese patients with SAB suggest that continued use of P2Y12 inhibitors may be associated with a potentially low rate of persistent bacteremia compared to their non-use. This finding suggests a potential beneficial role of P2Y12 inhibitors in enhancing bacterial clearance in SAB. There was no significant difference in the 30-day or in-hospital mortality rates between P2Y12 inhibitor users and non-users, but the absence of deaths among P2Y12 inhibitor users is of interest. Furthermore, the incidence of bleeding events did not increase among P2Y12 inhibitor users during hospitalization, indicating a favorable safety profile.

Previous studies have shown that P2Y12 inhibitors may be repurposed as adjunct therapies to improve the outcomes of patients with SAB [8,9]. A national retrospective study in the United States showed that inpatient mortality (hazard ratio (HR): 0.11, 95% confidence interval (CI): 0.01-0.86) and 30-day mortality (HR: 0.43, 95% CI: 0.19-0.98) rates were significantly lower among clopidogrel users than in non-users. However, they found no differences in microbiological clearance [8]. In contrast, our study did not show a significant difference in mortality, although there were no deaths among P2Y12 inhibitor users. Interestingly, we observed a significant improvement in microbiological clearance among P2Y12 inhibitor users, which aligns with a case report describing successful clearance of bacteremia with ticagrelor administration in a patient with persistent bacteremia even after receiving proper antimicrobial treatment for *S. aureus* endovascular infection [9]. The timing of P2Y12 inhibitor use in our study, which was prior to SAB onset, is consistent with the Veterans Affairs retrospective surveillance [8]. The previous study and our study focused on patients who were using P2Y12 inhibitors for at least 30 days prior to admission and continued their use for at least five days after admission. This continued use allowed the evaluation of potential protective effects against the development and progression of severe infections. P2Y12 inhibitors, through their anti-inflammatory and immunomodulatory effects [3-6], may create a less favorable environment for bacterial growth even before the onset of infection, potentially leading to less severe presentations of SAB or reduced complications. These discrepancies in findings could be attributed to several factors, including differences in study populations, P2Y12 inhibitor use patterns, or potential differences in *S. aureus* strains. Our study focused on the Japanese population, known to have a higher prevalence of CYP2C19 polymorphisms than Caucasians [10]. Despite the genetic differences between our Japanese cohort and the predominantly Caucasian population in the Veterans Affairs study [8], we observed some similar benefits of P2Y12 inhibitor use in SAB treatment. This similarity could be due to several factors. First, the mechanisms by which P2Y12 inhibitors improve outcomes in SAB may not solely depend on their antiplatelet effects. Other properties, such as their anti-inflammatory actions, might play a major role and may be less affected by CYP2C19 polymorphisms. Second, the effect of CYP2C19 polymorphisms on P2Y12 inhibitor efficacy might be less pronounced in the context of SAB than their well-established effects in cardiovascular disease prevention. Third, in our patient population of chronic P2Y12 inhibitor users, steady-state drug levels might have been achieved that are sufficient to provide benefit, even in the presence of polymorphisms affecting drug metabolism. However, our study did not directly assess CYP2C19 genotypes or measure drug levels, limiting our ability to draw definitive conclusions regarding the effect of genetic polymorphisms on drug efficacy in this context.

It is crucial to acknowledge the significant differences in baseline characteristics between P2Y12 inhibitor users and non-users in our study. P2Y12 inhibitor users were more likely to be male individuals and have comorbidities associated with atherosclerotic disease, including diabetes mellitus, ischemic heart disease, and peripheral artery disease. These differences, identified through univariate analysis, are consistent with the primary indications for P2Y12 inhibitor use but necessitate careful consideration when interpreting our results. The underlying difference in cardiovascular risk profiles between the groups could potentially influence SAB outcomes. Interestingly, despite the generally higher risk of complications such as infected aneurysms in patients with atherosclerosis [13], we observed no such cases in the P2Y12 inhibitor group. We also noted differences in the distribution of infection sites between the groups. Skin and soft tissue infections were more common among P2Y12 inhibitor users, while infectious endocarditis was only observed in the non-user group. The absence of infectious endocarditis in the P2Y12 inhibitor group aligns with previous reports suggesting a potential protective effect of antiplatelet agents against infectious endocarditis [14,15]. However, the small cohort and the inability to perform multivariate analysis due to cohort size limitations preclude definitive conclusions from these observations. These differences in baseline characteristics and infection sites underscore the complexity of interpreting our results and highlight the need for larger studies with more balanced groups to better control for these potential confounding factors.

The potential role of P2Y12 inhibitors in SAB treatment is highlighted by our finding of improved microbiological clearance, which is particularly relevant in the context of treating elderly patients. This population, accounting for a large portion of our study cohort, is known to have a higher incidence of SAB [16] and typically exhibits higher mortality rates [17]. Notably, we observed no deaths among P2Y12 inhibitor users, suggesting potential benefits in this vulnerable group. However, the therapeutic benefits must be carefully balanced against risks, particularly in elderly patients. While we observed no bleeding events during our study, a comprehensive meta-analysis has indicated an increased risk of major bleeding with P2Y12 inhibitor use in elderly individuals [18].

Recent studies have shown that different P2Y12 inhibitors may have varying effects on SAB treatment. For instance, ticagrelor has been shown to enhance platelet-mediated killing of *S. aureus*, possibly by protecting platelets from alpha-toxin-mediated injury and promoting clearance [7,9]. Although we primarily used clopidogrel, it may have similar effects. Interestingly, retrospective studies have demonstrated that patients taking ticagrelor had significantly fewer hospital readmissions due to infection, lower rates of gram-positive infection, and fewer episodes of SAB than those taking clopidogrel [19-21]. While our study did not include ticagrelor users, these findings underscore the need for further research to determine the efficacy of different types of P2Y12 inhibitors in SAB treatment, particularly in elderly populations. Future large-scale prospective studies should focus on the optimal timing of P2Y12 inhibitor administration, comparing prior use to initiation at the time of infection diagnosis, and evaluating the efficacy and safety profiles of different P2Y12 inhibitors across various age groups.

This study has some limitations. The most important limitation is the substantial imbalance between the number of P2Y12 inhibitor users (n=19) and non-users (n=219). While this disparity reflects the real-world distribution of P2Y12 inhibitor use in our SAB patient cohort, it significantly affects the statistical power and generalizability of our findings. The small number of P2Y12 inhibitor users limits our ability to detect small to moderate differences between groups and results in less precise estimates for this group, as evidenced by wider CIs. This imbalance, coupled with the overall small cohort, prevented us from performing multivariate analysis to adjust for potential confounding factors. Consequently, our results should be interpreted cautiously, as unmeasured confounders may have influenced the observed associations. The retrospective, single-center nature of our study introduces potential biases and increases the variability of results by chance, reducing the reproducibility of our findings. Our study design, which included only patients already taking P2Y12 inhibitors before admission, may have introduced selection bias. During antimicrobial therapy, P2Y12 inhibitor users were required to remain on medication, potentially excluding severely ill patients who had discontinued chronic medication at admission. This could have led to an underestimation of adverse outcomes in the P2Y12 inhibitor group. Additionally, our assessment of bleeding risk was limited to the hospitalization period, providing insufficient data on long-term bleeding risk associated with P2Y12 inhibitor use. Despite these limitations, our study provides important preliminary data on the potential benefits of P2Y12 inhibitors in SAB treatment, particularly regarding microbiological clearance.

## Conclusions

Our study demonstrated the potential benefits of P2Y12 inhibitors as adjunctive therapy in SAB treatment, particularly in the Japanese population. The significant improvement in microbiological clearance among P2Y12 inhibitor users suggests their role in enhancing bacterial elimination. However, the lack of significant differences in mortality rates and potential risks, especially in elderly patients, necessitates careful consideration of the risk-benefit profile. Although our study significantly contributes to the growing evidence supporting the repurposing of P2Y12 inhibitors, large prospective studies are needed to establish the efficacy and safety of P2Y12 inhibitors in SAB treatment and to elucidate the underlying mechanisms of their effects.
